# Ending HIV, hepatitis B, and hepatitis C: what about people with severe mental illness?

**DOI:** 10.1016/S2215-0366(17)30282-1

**Published:** 2017-07-04

**Authors:** Milton Wainberg, Lisa Dixon

**Affiliations:** Columbia University Medical Center, New York State Psychiatric Institute, New York, NY 10032, USA

HIV, hepatitis B (HBV), and hepatitis C (HCV) are preventable serious blood-borne infections. Early detection and accessible and user-friendly treatments improve prognosis, cure (for HCV), and prevent further transmission. 30 years of previous studies in a range of population samples have suggested that severe mental illness is a risk factor for contracting blood-borne viruses (BBVs).^1^ In *The Lancet Psychiatry*, Clarissa Bauer-Staeb and colleagues^2^ confirm the elevated risk for BBVs experienced by individuals with severe mental illness in a total population study of Sweden, finding that the odds of HIV were 2·57 (95% CI 2·25–2·94, p<0·0001) times higher in people with severe mental illness than in the general population, whereas the odds of HBV were 2·29 (2·09–2·51, p<0·0001) times higher and the odds of HCV were 6·18 (5·98–6·39, p<0·0001) times higher. This result increases confidence in the validity of previous studies with more narrowly selected samples.

After the first case of HIV infection among people with severe mental illness was identified in 1983,^3^ multiple studies worldwide have reported high prevalence of HIV infection among people with severe mental illness.^1^ Since then, pioneering research in the USA with people with severe mental illness has shown positive effects on a range of outcomes including measures of condom use, HIV knowledge, and sexual behaviours^4^—yet the epidemic among people with severe mental illness continues to be inadequately addressed. High prevalence of HBV and HCV are also well known and are similarly unaddressed.^1^ Over the past 5 years, the increasingly well established disparities in the physical health of people with severe mental illness have become health policy priorities; however, these policies do not target sexual health and risk factors facilitating transmission of BBVs.^1^

Transmission of BBVs occur through unprotected anal, vaginal, or oral sex; through mother to child vertical transmission; by sharing drug-injecting paraphernalia; and parenterally with contaminated blood and blood products or contaminated instruments and needles. In some circumstances, people with severe mental illness might be more vulnerable to engaging in behaviours that increase their risk of infection with BBVs, including unprotected sex, sex in exchange for a commodity (eg, money, shelter, food), and sharing equipment for intravenous drug use. Ending the HIV epidemic requires routine testing for HIV infection; prevention of infection among HIV-negative individuals with effective risk-reduction interventions and prescriptions of pre-exposure prophylaxis; and antiretroviral treatment for individuals who are HIV-positive until viral suppression is accomplished (treatment as prevention) to decrease forward transmission.^5^ A vaccine against HBV confers greater than 95% immunity in three doses.^6^ Screening for HCV followed by treatment can cure infection in more than 95% of patients.^7^ One question raised by Bauer-Staeb and colleagues’ Article^2^ is whether individuals with a diagnosis of severe mental illness have as much access to these interventions as do people without severe mental illness.

Education alone does not confer prevention. Routine HIV, HBV, or HCV testing has not been implemented in most mental health settings. Efficacious HIV and HCV risk-reduction interventions are available; however, they are either not offered in psychiatric settings or, when they are, they are offered only to those known to be HIV-infected or who self-disclose risky sexual behaviour. People whose risks are not so obvious are easily overlooked. Similarly, HBV vaccination and HCV treatment are seldom available in mental health-care settings. The inaccurate assumption made by some mental health providers that people with severe mental illness do not engage in intravenous drug use or unprotected sex can result in patients not receiving proper care.^1,8^

Psychiatric facilities are perfect settings for implementation of testing, prevention, and treatment of BBVs tailored to individuals with severe mental illness.^9^ Caring and supportive providers already engaged in therapeutic relationships with their patients can address barriers, alleviate concerns, and facilitate comprehensive prevention and care. Barriers to provision of prevention, testing, and care to people with severe mental illness include insufficient training of mental health staff in evidence-based interventions; inadequate funds, including little money for condoms; the separation of psychiatric and medical services; and providers’ failure to address patients’ romantic partnerships, sexual needs, and psychotropic medication side-effects.^9^

Global efforts to end HIV, HBV, and HCV as a public health threats are underway, but the extent to which public psychiatric care systems are participating is unknown despite evidence that individuals with severe mental illness have higher prevalence of BBVs than those in the general population. Recovery guidelines include integration of physical health and mental health care,^10,11^ but uptake of prevention and intervention strategies for HIV, HBV, and HCV has been scarce in real-world mental health settings.^12^ People with severe mental illness must be specifically included in current well funded global initiatives to reduce transmission of BBVs and achieve the goal of ending the HIV, HBV, and HCV epidemics.^5^ This goal requires a greater commitment from governments and public mental health-care systems to systematically and comprehensively reach this disproportionally affected population.

## References

[1] Hughes E, Bassi S, Gilbody S, Bland M, Martin F. Prevalence of HIV, hepatitis B, and hepatitis C in people with severe mental illness: a systematic review and meta-analysis. Lancet Psychiatry 2016; 3: 40–48. 26620388 10.1016/S2215-0366(15)00357-0 PMC4703902

[2] Bauer-Staeb C, Jörgensen L, Lewis G, Dalman C, Osborn DPJ, Hayes JF. Prevalence and risk factors for HIV, hepatitis B, and hepatitis C in people with severe mental illness: a total population study of Sweden. Lancet Psychiatry 2017; published online July 4. 10.1016/S2215-0366(17)30253-5. PMC5573766 28687481

[3] Cournos F, Empfield M, Horwath E, HIV seroprevalence among patients admitted to two psychiatric hospitals. Am J Psychiatry 1991; 148: 1225–30. 1883002 10.1176/ajp.148.9.1225

[4] Pandor A, Kaltenthaler E, Higgins A, Sexual health risk reduction interventions for people with severe mental illness: a systematic review. BMC Public Health 2015; 15: 138. 25886371 10.1186/s12889-015-1448-4 PMC4330652

[5] Piot P, Karim SSA, Hecht R, Defeating AIDS—advancing global health. Lancet 2015; 386: 171–218. 26117719 10.1016/S0140-6736(15)60658-4

[6] WHO. Hepatitis B. Fact sheet http://www.who.int/mediacentre/factsheets/fs204/en/(accessed July 3, 2017).

[7] WHO. Hepatitis C. Fact sheet http://www.who.int/mediacentre/factsheets/fs164/en/ (accessed July 3, 2017).

[8] Yehia BR, Cui W, Thompson WW, HIV testing among adults with mental illness in the United States. AIDS Patient Care STDS 2014; 28: 628–34. 25459230 10.1089/apc.2014.0196 PMC4250950

[9] Wainberg ML, González MA, McKinnon K, Targeted ethnography as a critical step to inform cultural adaptations of HIV prevention interventions for adults with severe mental illness. Soc Sci Med 2007; 65: 296–308. 17475382 10.1016/j.socscimed.2007.03.020 PMC2175036

[10] Patel V, Araya R, Chatterjee S, Treatment and prevention of mental disorders in low-income and middle-income countries. Lancet 2007; 370: 991–1005. 17804058 10.1016/S0140-6736(07)61240-9

[11] Pincus HA, Spaeth-Rublee B, Sara G, A review of mental health recovery programs in selected industrialized countries. Int J Mental Health Syst 2016; 10: 73. 10.1186/s13033-016-0104-4 PMC5131415 27956939

[12] Whiteman KL, Naslund JA, DiNapoli EA, Bruce ML, Bartels SJ. Systematic review of integrated general medical and psychiatric self-management interventions for adults with serious mental illness. Psychiatr Serv 2016; 67: 1213–25. 27301767 10.1176/appi.ps.201500521 PMC5089924

